# Unpacking postpartum depression in rural India: an integrated analysis of risk factors at 12 months and child development outcomes at 18 months of age – findings from the SPRING study

**DOI:** 10.1186/s40359-025-03746-1

**Published:** 2026-01-19

**Authors:** Divya Kumar, Seyi Soremekun, Reetabrata Roy, Sunil Bhopal, Deepali Verma, Kamal Kant Sharma, Neha Batura, Gauri Divan, Betty Rosamund Kirkwood, Bilal Iqbal Avan

**Affiliations:** 1 Department of Population Health, Faculty of Epidemiology & Population Health, London School of Hygiene & Tropical Medicine, London, United Kingdom; 2 Child Development Group, Sangath, Delhi, India; 3 Department of Infection Biology, Faculty of Infectious and Tropical Diseases, London School of Hygiene and Tropical Medicine, London, United Kingdom; 4 Bradford Institute for Health Research, Bradford, United Kingdom; 5 Global Business School for Health, University College London, London, United Kingdom

**Keywords:** Postpartum depression, Maternal mental health, Risk factors, Early child development, Developmental delays, Rural india, Longitudinal study

## Abstract

**Background:**

Postpartum depression (PPD) negatively affects maternal well-being and early child development (ECD). While research often focuses on the early postpartum depression, symptoms can emerge or persist later in the first year, potentially affecting mothers and children differently at this stage. Yet, the 12-month postpartum period remains understudied globally, particularly in low-resourced settings where contextual stressors are pronounced. This study addresses the urgent need to understand later-stage postpartum depression and how it influences child development at 18 months, a critical period for identifying early developmental delays.

**Methods:**

We analysed longitudinal data from a cluster randomised trial of an integrated mother-child intervention in rural India (Haryana). Mother-child dyads enrolled between 18 June 2015 and 1 July 2017 were assessed for PPD at 12 months using Patient Health Questionnaire-9 (PHQ-9) and for child development at 18 months with Bayley Scales of Infant and Toddler Development-III. We examined PPD risk factors using logistic regression and the association between PPD and ECD through multinomial regression models, accounting for covariates and clustering.

**Results:**

Among 2007 mothers assessed at 12 months, PPD prevalence was 13.1% (PHQ-9 ≥ 5). Maternal adverse events (OR = 1.53, CI: 1.35–1.73, *p* < 0.001) and psychosocial stress (OR = 1.10, 95% CI: 1.08–1.12, *p* < 0.001) increased PPD odds, while psychosocial support (OR = 0.97, 95% CI: 0.96–0.98, *p* < 0.001) and higher socio-economic status (OR = 0.72, 95% CI: 0.64–0.79, *p* < 0.001) reduced them. Among 1250 dyads assessed at 18 months, PPD predicted mild-to-moderate (RRR = 1.63, 95% CI: 1.13–2.36, *p* < 0.01) and severe (RRR = 1.64, 95% CI: 1.03–2.61, *p* < 0.05) language delays, but not cognitive or motor delays. These associations were not significant after adjustment.

**Conclusions:**

This is among the first Indian studies to longitudinally examine the association between postpartum depression at 12 months and child development at 18 months, addressing a major evidence gap in rural settings. The study identified a distinct risk profile for postpartum depression shaped by socioeconomic and maternal adversity. Postpartum depression showed domain-specific associations with language delays, explained by contextual factors. These findings highlight the need to integrate evidence-based mental health screening and support for at-risk mothers and children into existing maternal and child health services in rural contexts.

**Supplementary Information:**

The online version contains supplementary material available at 10.1186/s40359-025-03746-1.

## Introduction

Postpartum depression (PPD) remains a pressing public health issue, with well documented effects on mothers and children, though this relationship is not entirely straightforward. Socioeconomic, psychosocial, and contextual factors all appear to shape this dynamic [1, 2]. In low-resource settings, risks like poverty, intimate partner violence (IPV), and lack of social support have been recognised to compound vulnerability to PPD [3, 4].

In India, the evidence on PPD reveals critical gaps. A recent meta-analysis of Indian studies reported a 22% overall pooled prevalence of PPD, with lower rates observed in rural areas (17%, SD) compared to urban areas (24%) [5]. Regional studies, including Karnataka, Tamil Nadu, Kolkata and Goa identify four recurrent risk factors: financial insecurities, inadequate social support networks, IPV, and son preference [5, 6], while nuclear households has been identified as a predictor by few [5]. Although, these findings offer valuable insights, the literature remains fragmented, and few longitudinal, rural focused studies comprehensively examine how socioeconomic and cultural contexts, influence maternal vulnerability to PPD in distinct ways.

Despite growing recognition that chronic PPD can affect maternal well-being, caregiving, and prolong her child’s exposure to depressive symptoms [7, 8], few studies have assessed PPD beyond the early postpartum period. Notably, only two Indian studies, have captured the 12-month PPD prevalence and risk factors [9, 10], highlighting a critical underexplored area in this literature.

The gap extends to PPD’s impact on early child development (ECD). Most research globally, including in India, has focused on the first year [8, 11]. However, to understanding PPDs longer-term consequences requires child development assessments beyond the first year of life, as delays may not be fully detectable at earlier ages [12]. Eighteen months marks a critical developmental stage, as children begin walking, using language, engaging socially, and making sense of the world through play and everyday objects [13]. Yet, few studies extend follow-up to this age [7, 8]. In India, national surveys mostly report aggregated under-five indicators [14], obscuring age-specific patterns. Evidence from Indian studies remains limited, mixed across development domains [15], and rarely focuses on rural settings or extend beyond infancy. To our knowledge, only one study from a comparable low- and middle- income country (LMIC) setting Pakistan [16], has examined this association at 18 months of age.

These inconsistencies point to the need for multi-level approaches to understand how local risk factors shape both PPD and its developmental consequences, especially in underrepresented rural LMIC settings [2]. While most studies examine either what contributes to PPD or whether it affects ECD, few adopt an integrated approach that treats PPD both as an outcome of multiple risks and, subsequently, as a potential risk factor for developmental delays, traced across distinct time points within the same cohort. The present study takes this dual perspective, addressing two interrelated questions: (1) What are the primary risk factors for PPD at 12 months in rural India? and (2) Does PPD influence child development outcomes at 18 months, and do any of the identified risk factors help account for this association?

By doing so, this study offers a rare longitudinal view of a later postpartum period, placing mother’s mental health at the centre of inquiry, not only as an outcome but also as a potential driver of her child’s early developmental trajectory.

## Materials and methods

### Overview of study design

This study is based on longitudinal data collected as part of the Sustainable PRogramme Incorporating Nutrition and Games (SPRING) cluster randomized controlled trial (cRCT). The trial evaluated an integrated community-based mother–child intervention in rural India to assess its relationship with child development by improving maternal well-being, specifically PPD, as a secondary outcome (details published elsewhere [17]; see intervention’s brief description in Supplementary File 1). While the primary trial was designed to evaluate intervention effectiveness, our particular analysis broadened the focus to longitudinal relationships within the trial cohort of mother-child dyads. Drawing on its rich and systematically collected data, we examine both risk factors and developmental consequences of PPD across the 12–18 month postpartum period (see Analytical Framework, Supplementary File 2). The analysis accounts for the cluster-randomized design, trial arm allocation, and potential confounders as detailed in the analysis section ahead.

### Study setting

The study was conducted in 120 predominantly farming-based villages across three administrative blocks in Rewari district, Haryana, in northern India. As per the 2011 Census, Rewari’s population was just over 900,000, with 74% in rural areas. Literacy rates were relatively high (male: 91%, female: 69%) versus state (male: 84.1%, female: 65.9%) and national average (male: 80.89%, female: 64.64%). However, the district reported one of Haryana’s lowest sex ratios of 787 per 1,000 in the 0–6 age group, well below state and national levels. Institutional delivery coverage was 99%, surpassing state (95%) and national (89%) levels [18, 19]. The study was embedded within this broader context of social progress and persistent gender disparities.

### Study participants

Participants were mother–child dyads with live births identified through a project-established, house-to-house surveillance system between 18 June 2015 and 1 July 2017 and enrolled into the SPRING trial [17]. For PPD risk factors analysis, the cohort included 2007 mothers who completed the PPD assessment at 12 months postpartum. For the analysis of the association between PPD and child development, we included mother–child dyads with complete data for both: maternal PHQ-9 assessments at 12 months and child BSID-III assessments at 18 months, yielding an analytic sample of 1,250 dyads. Exclusion criteria were major congenital malformations, maternal death during follow-up, child death by 12 months, and inability of the mother to participate in interviews.

### Sample size

The sample size calculations for the original cluster trial, detailed elsewhere [17], accounted for evaluation of both intervention effects on PPD and its subsequent impact on child development at 18 months. The current analysis, drawing on the same cohort, addresses part of this causal pathway by examining PPD risk factors and PPD-ECD associations. This observational analysis approach compares mothers with and without PPD in naturally occurring groups, independent of intervention effects.

To confirm statistical power for the risk factor analysis, we conducted verification using G*Power [20] drawing on effect size estimates from LMIC studies [5, 8]. From the range reported, we selected the smallest detectable odds ratio (OR) for the first risk factor to avoid overestimation of power. Assuming a 22% baseline PPD prevalence (as per a meta-analysis of Indian studies) [5], a sample size of 2,007 yields 82.46% power at α = 0.05 to detect an OR of 1.6 [21, 22]. These calculations were based on procedures proposed by Hsieh [23] and Demidenko [20]. Adjustments were made for additional risk factors (covariates) in a multivariate model using an R² value of 0.16, calculated based on inter-covariate correlations.

For the PPD–ECD analyses, similar power calculations were conducted. Assuming a 16% prevalence of developmental delays in the population [24], and a sample of 1,250 dyads, we achieve 88.34% power to detect an OR = 1.8 [8, 25] at a 5% significance level in a comparison of the risk of developmental delay in infants of mothers with, and without PPD. This analysis incorporated a covariate adjustment with R² = 0.06.

In sum, we used two analytic samples drawn from the same cohort: one all mothers for the PPD risk factors (*n* = 2,007), and two, mother-child dyads for PPD-ECD association (*n* = 1,250). The conservative power assumptions, along with the prospective design of the original cluster trial and its secondary analyses, strengthen the analytical rigour of this study.

### Outcome measures

#### PHQ-9

 PPD was assessed at 12 months postpartum using the Patient Health Questionnaire-9 (PHQ-9), a validated 9-item screening tool aligned with Diagnostic and Statistical Manual - Version IV (DSM-IV) criteria for depression [26]. Individual items are scored from 0 to 3, yielding a total score between 0 and 27. Scores ≥ 5 indicate depression. PHQ-9 was selected for its brevity, suitability for low-literacy settings [27, 28], and Hindi validation for use in India [29].

#### BSID-III

 Child development outcomes were assessed at 18 months using the Bayley Scales of Infant and Toddler Development, Third Edition (BSID-III) [30], measuring cognitive, language (receptive and expressive), and psychomotor (gross and fine motor) domains. The scales were culturally adapted for the local context through forward-translation, cognitive testing, and piloting [17, 31].

#### Potential risk factors for PPD

Variables identified from baseline data and existing literature were grouped into three risk categories and presented in Table 1: (1) demographic and economic, (2) psychosocial support and stress, and (3) maternal adverse events (MAE).

**Table 1:** Definitions of risk factors for 12-Month PPD in rural India

	Determinants/Risk Factors	Type of variable and definition
A.	Demographic and Economic	
1.	Maternal age at childbirth	Continuous (age range: 15–49 years)
2.	Maternal education	Categorical: 0 = > 5 years of education completed; 1 = no or ≤5 years of education;
3.	Mother’s socio-economic status (SES)	Categorical variable in quintiles (1–5); 1 = poorest (bottom 20%), 5 = least poor (top 20%)
4.	Mother’s Caste	Categorical: 0 = general category; 1 = scheduled caste, scheduled tribe or other backward class (SC/ST/OBC)
5.	Place of delivery	Categorical: 0 = facility-based (home, midwife’s residence, enroute); 1 = non-facility-based (private hospital, health subcentre, primary health centre, community health centre, district/civic hospital)
6.	Gender of the child	Categorical: 0 = male; 1 = female
B.	Mother’s psychosocial support and stress	
7.	Duke social support score	Continuous: Support from all people (11 item tool, 22 points)
8.	Duke family support score	Continuous: Support from family members (maximum 7 items, 14 points)
9.	Duke non-family support score	Continuous: Support from non-family members (maximum 5 items, 10 points)
10.	Duke Stress score	Continuous: Stress from all people (11 item tool, 22 points)
11.	Duke family stress score	Continuous: Stress from family members (maximum 7 items, 14 points)
12.	Duke non-family stress score	Continuous: Stress from non-family members (maximum 5 items, 10 points)
C.	Maternal adverse events (MAE)	
13.	MAE	Continuous variable: An additive index (0–7) created by summing stressful life events from pregnancy to 12 months postpartum, based on Early Life Stress Questionnaire [32]. Events included: (1) serious maternal illness, (2) illness or death of a close family member, (3) husband’s alcohol-related problems, (4) physical abuse by husband, (5) verbal abuse by husband, (6) mistreatment by other family members, and (7) family indebtedness.

### Data collection

PHQ-9 was administered with mothers at 12 months postpartum, and the BSID-III administered to their infants at 18 months postpartum (− 7 to + 21 days from the child’s 18-month birthday). A dedicated outcome assessment team—locally recruited and trained—administered the PHQ-9 at the 12-month visit as part of a broader set of maternal and household assessments. The PHQ-9 itself was brief, typically taking about 5 min to complete. At 18 months, the same team administered the BSID-III, which required two assessors and approximately 1–1.5 h per child. Data quality was ensured through routine and booster trainings, enhanced supervision, and regular accuracy and completeness checks.

### Ethical considerations

Ethical approvals were obtained from the London School of Hygiene & Tropical Medicine (SPRING: 23 June 2011, 5983; secondary analysis: 14 November 2017, 14445), the Sangath Institutional Review Board, India (SPRING: 19 February 2014), and the Indian Council of Medical Research’s Health Ministry Screening Committee (SPRING: 24 November 2014). All participants provided informed consent at the enrolment stage and the beginning of the outcome assessment phase.

### Statistical Methods/Data analysis

Analyses were conducted in two parts using STATA 18 (StataCorp LLC: College Station, TX, USA).

#### Part 1: risk factors for PPD at 12 months after childbirth

Potential risk factors for PPD were analysed among all mothers who completed the PHQ-9 at 12 months. Mixed-effects logistic regression was used in the final multivariable model to estimate the binary outcome (presence or absence of PPD), accounting for trial arm allocation and clustering effects. Initially, univariate ORs with 95% confidence intervals and p-values were estimated for each variable. These were grouped into three predefined categories (Table 1). An additive index for MAE was constructed based on theoretical relevance and variables identified as significant in univariate analysis. Variables with *p* < 0.05 in univariate models were retained for multivariable analysis. In addition, some variables identified a priori as relevant based on existing literature (e.g., maternal education, child’s gender) were included regardless of statistical significance.

#### Part 2: association between PPD and ECD outcomes

The association between PPD and child development at 18 months was analysed among mother–child dyads with complete data at both 12 and 18 months. Developmental outcomes were classified into three categories: (1) no delay, (2) mild-to-moderate delay (composite score ≥70 and < 85; between − 1 SD and − 2 SD of the global mean), and (3) severe delay (< 70; below − 2 SD), based on BSID-III guidelines [30]. Multinomial logistic regression models were fitted with PPD as the binary predictor. Results are reported as relative risk ratios (RRRs), comparing outcomes for children of mothers with and without PPD. The final model adjusted for trial arm, village clustering, and a set of key covariates identified as significant risk factors.

## Results

### Participant flow

**Fig. 1:**
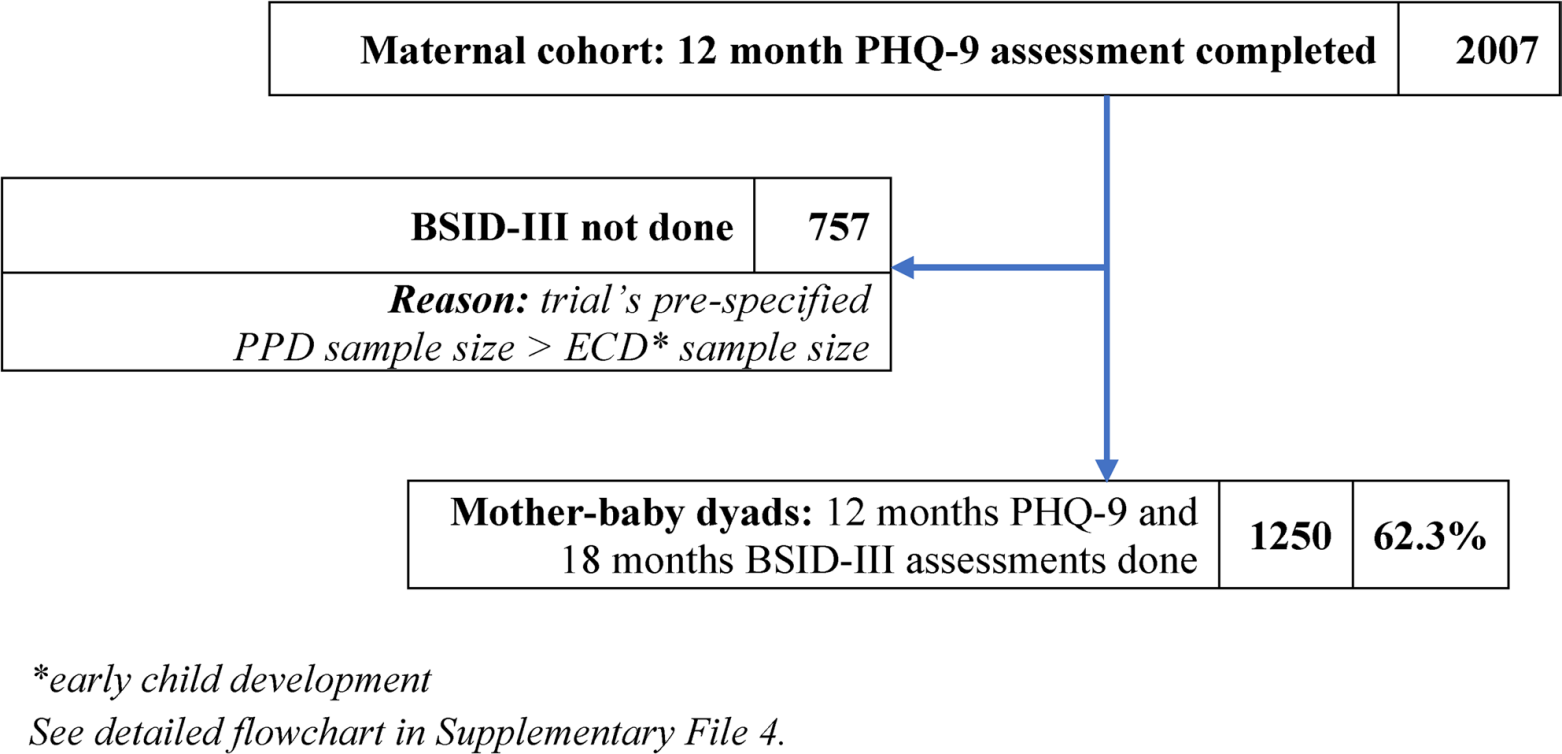
Participant flow for analysis of 12-month PPD and 18-month child development outcomes

Figure 1 shows the participant flow from the 12-month outcome assessments to the 18-month timepoint. A total of 2,007 mothers completed the 12-month outcome assessments, forming the initial maternal cohort for this paper’s analysis of risk factors. Of these, 1,250 mother–child dyads contributed data at 18 months – planned in the parent trial (explained below) and with its pre-specified sample size calculations. Importantly, the 1,250 dyads exceeded the originally planned minimum sample size for PPD–ECD analyses (1,200). This analytic sample was primarily based on protocol-driven requirements for complete data, rather than losses due to follow-up, thereby minimising risk of bias. For maternal outcomes (e.g., PHQ-9), each mother was counted once, regardless of whether she had twins; for child development outcomes (BSID-III), all her children were assessed, including twins. Cronbach’s α values for outcome measures used in this analysis are presented in Supplementary File 3.

Inclusion in the PPD-ECD analysis followed a predefined, birth-timing based selection process in the main trial, with the first 80 mothers per cluster reaching 12 months eligible for PPD assessment and the first 50 reaching 18 months eligible for developmental assessment. To minimise systematic differences, this criterion was applied uniformly across clusters to ensure that inclusion was independent of maternal or household characteristics. Supplementary File 5 presents the descriptive statistics for mothers with a completed PHQ-9 assessment, and amongst these, compares mothers who were included in the PPD–ECD part of the analysis with those excluded. This allows an assessment of how similar the two groups of mothers were and helps identify any potential systematic differences, thereby supporting transparency in reporting.

####  Part 1: risk factors for PPD at 12 months after childbirth

The overall prevalence of PPD (PHQ-9 ≥ 5) at 12 months postpartum was 13.1% among 2007 mothers. As shown in Table 2, maternal age, education, child’s gender, facility-based birth, and family size were not significantly associated with PPD. Socioeconomic status (SES), however, showed a significant inverse relationship with PPD - 34% of mothers with PPD were in the lowest SES quintile versus 8% in the highest, with each quintile increase linked to 28% lower odds of PPD (OR = 0.72, 95% CI: 0.64–0.79, *p* < 0.001).

**Table 2:** Risk factors for PPD at 12 months postpartum in rural India: univariate analysis

S. No.	Risk factors		Total	With PPD	Without PPD		OR (95% CI)		*p*-value	
			2007	262	1745					
	Demographic and economic									
1.	Maternal education *n* (%)	<=5years	220 (10.97%)	35 (13.36%)	185 (10.61%)		1.19 (0.80, 1.77)		0.379	
		5 + years	1786 (89.03%)	227 (86.64%)	1559 (89.39%)					
3.	Mothers SES (Quintiles) *n* (%)	Quintile 1	412 (20.53%)	86 (32.82%)	326 (18.68%)		0.72 (0.65, 0.79)		< 0.001	
		Quintile 2	419 (20.88%)	70 (26.72%)	349 (20.00%)					
		Quintile 3	404 (20.13%)	44 (16.79%)	360 (20.63%)					
		Quintile 4	406 (20.23%)	41 (15.65%)	365 (20.92%)					
		Quintile 5	366 (18.24%)	21 (8.02%)	345 (19.77%)					
8.	Mother’s giving birth to a female child *n* (%)	Yes	898 (44.74%)	125 (47.71%)	773 (44.30%)		1.13 (0.89, 1.42)		0.295	
		No	1109 (55.26%)	137 (52.29%)	972 (55.70%)					
10.	Mother’s belonging to lower caste category *n* (%)	Yes	1232 (61.39%)	156 (59.54%)	1076 (61.66%)		1.01 (0.78, 1.30)		0.946	
		No	775 (38.61%)	106 (40.46%)	669 (38.34%)					
12.	Place of childbirth *n* (%)	Non-facility	38 (1.89%)	4 (1.53%)	34 (1.95%)		0.78 (0.27, 2.26)		0.656	
		Facility	1969 (98.11%)	258 (98.47%)	1711 (98.05%)					
14.	Maternal age at childbirth (in years)	Mean (sd)	22.32 (3.84)	22.24 (3.90)	22.33 (3.83)		0.99 (0.96, 1.03)		0.945	
15.	Family Size	Mean (sd)	6.23 (2.63)	6.15 (2.88)	6.24 (2.60)		0.98 (0.93, 1.03)		0.527	
	Mother’s psychosocial support and stress									
16.	Overall social support	Mean (sd)	62.2% (12.20)	58.1% (14.73)	62.8% (11.64)		0.97 (0.96, 0.98)		< 0.001	
17.	• Family support subscale	Mean (sd)	83.8% (14.58)	76.8% (17.8)	84.8% (13.72)		0.97 (0.96, 0.98)		< 0.001	
18.	• Non-family support subscale	Mean (sd)	37.9% (14.33)	37.2% (15.53)	37.9% (14.14)		0.99 (0.98, 1.00)		0.536	
19.	Overall social stress	Mean (sd)	2.4% (6.34)	6.87% (9.38)	1.77% (5.44)		1.10 (1.08, 1.12)		< 0.001	
20.	• Family stress subscale	Mean (sd)	3.5% (9.41)	10.3% (14.52)	2.44% (7.89)		1.06 (1.04, 1.07)		< 0.001	
21.	• Non-family stress subscale	Mean (sd)	1.7% (5.66)	4.12% (8.65)	1.31% (4.95)		1.06 (1.04, 1.08)		< 0.001	
	Maternal adverse events (MAE)	Response	Overall n (%)	With PPD n (%)	Without PPD n (%)		OR (95% CI)		*p*-value	
			1250 ^a^	230	1020					
22.	MAE (Additive index variable) [Item definitions and coding approach are provided in Supplementary File 6]	0	562 (44.96%)	63 (27.39%)	499 (48.92%)		1.53 (1.35, 1.73)		< 0.001	
		1	402 (32.16%)	83 (36.09%)	319 (31.27%)					
		2	175 (14.00%)	45 (19.57%)	130 (12.75%)					
		3	68 (5.44%)	17 (7.39%)	51 (5.00%)					
		4	25 (2.00%)	9 (3.91%)	16 (1.57%)					
		5	14 (1.12%)	10 (4.35%)	4 (0.39%)					
		6	4 (0.32%)	3 (1.30%)	1 (0.10%)					

^a^ Data unavailable for one mother on education

^b^ The OR and *p*-value for quintile indicate a linear trend in the odds across increasing quintiles (1 to 5)

^c^ Adverse life events were assessed in a subsample of mothers (fewer than those with PHQ-9 data); all 1,250 had PHQ-9 assessments at 12 months and BSID-III at 18 months

*sd* standard deviation, *PPD* Postpartum depression

Psychosocial support and stress showed opposing trends (Table 2). Higher support was associated with lower odds of PPD (OR = 0.97, 95% CI: 0.96–0.98, *p* < 0.001), largely driven by family-based support while non-family support showed no association. In contrast, stress from any source significantly elevated PPD odds (OR = 1.10, 95% CI: 1.08–1.12, *p* < 0.001).

MAE showed a strong clear cumulative relationship with PPD. (Table 2). Each additional adversity raised the odds by 53% (OR = 1.53, 95% CI: 1.35–1.73, *p* < 0.001). Mothers with PPD were more likely to report multiple adversities: 9.5% had ≥ 4 events compared to 2.0% among non-depressed mothers. Although only a small number of mothers experienced ≥ 5 events, PPD prevalence was disproportionately higher in this group. Among individual adversities (Supplementary File 6), physical abuse (OR = 3.43) and verbal abuse (OR = 3.05) by the husband, and family mistreatment (OR = 3.46), each significantly tripling the odds of PPD (*p* < 0.001). These were followed by maternal illness and financial strain. Spousal alcohol problems showed a non-significant association.

In the adjusted model for PPD risk factors (Table 3), lower maternal SES, higher overall stress, and a greater number of adverse life events remained significantly associated with PPD. The association with social support, evident in univariate analysis, was attenuated after adjustment. No association was observed for child gender.

**Table 3:** Adjusted analysis - risk factors for PPD at 12 months in rural India

S. No.	Risk factors	Adjusted OR	95% CI	*p*-value
Demographic and economic risk factors				
1.	Mothers SES (Quintiles)	0.82	0.73, 0.92	0.001
2.	Child’s gender	1.02	0.75, 1.38	0.914
3.	Maternal education	0.62	0.38, 1.00	0.050
Mother’s psychosocial support and stress				
4.	Overall social support	0.99	0.98, 1.00	0.156
5.	Overall social stress	1.07	1.04, 1.09	< 0.001
Maternal adverse events (MAE)				
6.	MAE	1.29	1.13, 1.47	< 0.001

#### Part 2: association between PPD and ECD outcomes

Table 4 shows associations between maternal PPD and child development delays. Among children of mothers with PPD, 36.1% had language delays, and 12.2% had delays in both motor and cognitive domains compared to mothers without PPD.

**Table 4:** Association between PPD at 12 months and developmental delay in children at 18 months

Maternal PPD at 12 months after childbirth	Children without delay *n* (%)	Children with delay		−1SD delay (Mild – Moderate delay)		−2SD delay (Severe delay)	
		−1SD delay *n* (%)	−2SD delay *n* (%)	Non Adj RRR* (95% CI) *p*-value	Adj RRR (95% CI) *p*-value	Non Adj RRR (95% CI) *p*-value	Adj RRR (95% CI) *p*-value
Language delays at 18 M of child’s age: Association with maternal PPD at 12 months postpartum							
Overall	916 (73.28%)	215 (17.20%)	119 (9.52%)	1.63	1.37	1.64	1.46
With PPD	147 (63.91%)	53 (23.04%)	30 (13.04%)	1.13, 2.36	0.93, 2.02	1.03, 2.61	0.89, 2.36
Without PPD	769 (75.39%)	162 (15.88%)	89 (8.73%)	*p* = 0.009	*p* = 0.106	*p* = 0.038	*p* = 0.129
Motor delays at 18 M of child’s age: Association with maternal PPD at 12 months postpartum							
Overall	1129 (90.32%)	98 (7.84%)	23 (1.8%)	1.48	1.27	0.96	0.69
With PPD	202 (87.83%)	24 (10.43%)	4 (1.74%)	0.91, 2.42	0.76, 2.12	0.32, 2.87	0.22, 2.18
Without PPD	927 (90.88%)	74 (7.25%)	19 (1.86%)	*p* = 0.110	*p* = 0.350	*p* = 0.944	*p* = 0.532
Cognitive delays at 18 M of child’s age: Association with maternal PPD at 12 months postpartum							
Overall	1057 (84.56%)	162 (12.96%)	31 (2.48%)	0.77	0.68	0.45	0.32
With PPD	202 (87.83%)	25 (10.87%)	3 (1.30%)	0.49, 1.22	0.43, 1.10	0.14, 1.51	0.09, 1.12
Without PPD	855 (83.82%)	137 (13.43%)	28 (2.75%)	*p* = 0.277	*p* = 0.120	*p* = 0.196	*p* = 0.075

* *RRR* Relative Risk Ratio calculated, *Adj RRR* adjusted for maternal education, socio-economic status, child’s gender, and maternal adverse events

Maternal adverse events: additive index variable comprising seven adverse events (details in Supplementary File 6)

Developmental Delay: −1SD of global average (composite score ≥70 and < 85 on BSID-III); −2SD of global average (composite score < 70 on BSID-III)

*PPD* Postpartum depression

In unadjusted models, children of mothers with PPD had a 63% higher relative risk of mild-to-moderate language delays (RRR = 1.63, 95% CI: 1.13–2.36, *p* = 0.009) and a 64% higher risk of severe language delays (RRR = 1.64; 95% CI: 1.03–2.61; *p* = 0.038). After adjustment for demographic, socioeconomic, and adversity-related variables, these associations weakened and became non-significant, though estimates remained in the hypothesized direction (Table 4). No significant associations were observed for motor or cognitive delays. In additional analyses, both expressive and receptive language subdomains showed significant associations with PPD in unadjusted models, which attenuated after adjustment. Effect sizes were similar to those for overall language development.

## Discussion

Two key findings emerged from our analyses. First, socioeconomic and maternal adverse events were the dominant risk factors for PPD at 12 months. Second, PPD showed selective association with language delays at 18 months, and this association was shaped by contextual factors. We now examine each finding in detail.

### Risk factors for PPD

At 12 months postpartum, findings from rural India revealed a unique risk profile for PPD. While prior studies often emphasise social support as strongly protective, family adversity as moderately influential, and SES as only modestly associated [21, 33], our findings pointed to a different pattern. Psychosocial support showed unexpectedly weak associations, SES maintained a modest but consistent link, and maternal adversities emerged as the most prominent predictor. While partially overlapping with global and low-resource evidence [1, 5, 33], these findings highlight context-specific vulnerabilities for PPD in rural India.

The cumulative association between maternal adverse events and PPD highlights how layered stressors aggravate maternal mental health risks (Graph 1, Supplementary File 7). Among these, around one in every six to seven women with PPD reported physical or verbal abuse by their husband or mistreatment by other family members, compared to just one in twenty among non-depressed mothers. These specific adversities had particularly strong relationship, consistent with patterns reported across South Asia [1, 4, 34], Sub-Saharan Africa [1, 35], and Latin America [36]. This cross-regional consistency underscores how unequal gender roles and low maternal autonomy can heighten vulnerability to maternal mental health conditions. At the same time, contextual nuances matter: one Indian study found IPV more strongly linked to chronic depression following the birth of a daughter [4], reflecting how culturally embedded son preference can intensify the psychological toll of adversity. Spousal alcohol use, on the other hand, showed no associations in this setting, reinforcing prior evidence that PPD may be context-dependent or shaped by other underlying factors [37].

While family-based psychosocial support appeared protective in unadjusted models, its association with PPD did not hold after adjustment, contrary to evidence from other settings, where perceived (rather than received) support often shows stronger links [21, 38]. Psychosocial stress, especially interpersonal tensions, remained significantly associated with PPD in our sample, consistent with prior findings [1, 33]. This pattern may stem from the constraints of rural settings, where financial strain and caregiving responsibilities limit family’s ability to provide sustained practical support. Furthermore, depression-related cognitive distortions may also shape how mothers perceive the support and stress around them [39, 40]. One model suggests that stress may affect the relationship between support and PPD, with ongoing adversity potentially reducing its protective effects [41].

Economic disadvantage was associated with increased PPD risk, with a modest protective effect at higher SES levels (Graph 2, Supplementary File 7). Similar associations between poverty and maternal mental health have been documented across multiple settings [4, 33, 42]. Evidence from India suggests that financial interventions may reduce PPD risk. A quasi-experimental study of a conditional cash transfer programme reported a 36% reduction in PPD, likely by childbirth-related financial stress [43]. While further investigation is needed [42], this emphasises the value of integrating economic and psychosocial strategies for more comprehensive PPD prevention.

A notable departure from earlier South Asian studies was the absence of any observed child gender effect on PPD. Nearly 64% of Indian studies have reported increased PPD risk following the birth of a daughter [5], with similar findings in Turkey, China, and Japan [44]. In contrast, our results align with more recent evidence from India [45, 46] and Sub-Saharan Africa [3], suggesting growing variability in how child gender-related stress influences PPD within India. A study from India speculated that changing gender norms and girl-child welfare initiatives [47, 48] may be contributing to this shift [45], though these emerging pathways require further investigation.

While these findings concern commonly reported PPD risk factors, they reveal important contextual differences. The stronger influence of maternal adversity and the weaker-than-expected role of psychosocial support depart from earlier patterns, suggesting a distinct risk profile for PPD in the rural Indian context.

### Association between maternal PPD and ECD outcomes

Maternal PPD in this cohort was selectively associated with child development outcomes, specifically the language domain. Children exposed to PPD had higher relative risks of both mild-to-moderate and severe delays, though these attenuated after adjusting for socioeconomic and adversity-related factors, suggesting that broader contextual stressors, rather than PPD alone, may underlie these associations. Both expressive and receptive subdomains showed similar associations. While some researchers hypothesise a stronger effect on expressive language due to reduced maternal responsiveness, the evidence on these sub-domains remains limited and inconclusive [8, 49]. By contrast, no significant associations were observed with cognitive or motor development, indicating that PPD may not affect early development uniformly.

This initial association with language delays may be partly explained by how maternal depression disrupts verbal mother–child interactions. Previous studies show that depressed mothers often speak less, respond inconsistently, or withdraw emotionally, reducing opportunities for language stimulation [50]. In one Brazilian longitudinal study, persistent PPD across the first year was also associated with significantly lower language scores [51]. In rural India, these effects may be intensified by cultural expressions of distress. Depressive symptoms are often somatised, through backaches, headaches, or generalised body pain [52, 53]. Moreover, maternal withdrawal may take physical forms, such as lying down with a cloth covering their heads and eyes or avoiding interaction [52], possibly reducing verbal engagement during critical windows necessary for language development. These behaviours unfold within caregiving environments where child-directed speech is not commonly emphasised [54]. Moreover, caregiving is typically shared among family members and often prioritises compliance and quietness, with verbal expression less actively encouraged leading to child’s withdrawal from conversation [54, 55]. When PPD along with emotional withdrawal, is layered onto such caregiving styles, children may have even fewer opportunities for language use, increasing vulnerability to delay.

While certain caregiving behaviours may heighten vulnerability to language delays, they may simultaneously act as protective factors for other developmental domains. For example, distributed or multi-caregiver support may offer opportunities for physical exploration and non-verbal stimulation, which could better support motor and cognitive development [56]. Moreover, while such caregiving features may reduce the child’s exposure to maternal depressive symptoms, they may not offer the same protection for language development [54, 55], as explained above. Our finding of no association between PPD and cognitive or motor delays reflects the broader inconsistency in the global evidence. A prior review noted that only four of seven studies on motor, and three of eleven on cognitive development, did not report associations with PPD [8]. Some have suggested that PPD influenced development indirectly through caregiving quality [57] or an altered home environment rather than maternal mood alone [7, 8], with similar patterns observed for motor outcomes [8].

Further population-specific evidence may help explain selective developmental effects. The UK Millennium Cohort Study found lower odds of gross motor delays among Indian, Black African, and Black Caribbean infants compared to White infants, adjusted for socioeconomic factors [58]. Prior cross-cultural research observed rural Indian infants showing early motor advantages over US peers, though these declined by age two, possibly due to low birth weight [56, 59], as echoed by recent Indian studies that link developmental lags to stunting, highlighting the role of nutrition and physical health [12, 60].

Additionally, methodological differences, including symptom severity and timing, may explain discrepancies in findings. Studies with low PPD severity often reported null findings whereas adverse developmental outcomes were more evident in cases of chronic or severe PPD [8, 61]. Several LMIC studies [12, 16], including from India [15, 62], report early cognitive delays, particularly when both PPD and development are measured in the first year. Adverse effects were more likely when assessments occurred earlier (3–9 months), while studies with later assessments (12 months or beyond) often yielded null findings [12]. This suggests that short-term impacts may be more apparent even in milder cases, while longer-term effects are linked to persistent or severe depression. The present study differed from those reporting stronger associations, as PPD and child outcomes were each assessed only once, at two distinct time points, 12 and 18 months, respectively, and symptom severity in this sample was generally low. These factors may partly account for the weaker associations observed.

### Role of risk factors in PPD-ECD association

While PPD initially appeared associated with greater language delays at 18 months, this association did not persist after adjusting for socioeconomic and adversity-related factors - suggesting that observed relationship may be explained by broader contextual vulnerabilities co-occurring with maternal depression, rather than PPD alone [8, 16, 51]. This is supported by evidence from Pakistan, where the association between PPD and language delay (OR = 5.4) was evident only among the most economically disadvantaged households [16]. In our study too, mother’s SES emerged as a key independent correlate of language delay in fully adjusted models. Children from higher SES backgrounds showed better language performance, regardless of maternal PPD status, indicating that economic advantage may buffer early developmental risk [63]. This pattern suggests that developmental risks initially attributed to PPD, may in fact be more closely tied to structural disadvantage, including SES-related differences in caregiving quality [50, 63].

Extending this perspective, the enormity and diversity of maternal adversities in rural settings can influence both maternal mental health and early child development. Building on our earlier discussion of MAE as a risk factor for postpartum depression, evidence from other studies also points to broader maternal adversity as partly shaping early language outcomes. In this study, we tried to capture adversities that are specific to the rural context through MAE. Evidence from other studies also points to the role of broader maternal adversity in shaping early language outcomes. Exposure to abuse or violence against mothers by their partners in infancy has been linked with poorer expressive language, even in otherwise supportive and stimulating home environments. These associations often appear to be stronger for expressive than for receptive language [64]. Children exposed to anger, psychological aggression, or violence may become hesitant to speak or express themselves. Around age three, most children’s vocabularies grow rapidly, but in unsafe or unpredictable settings, they may hold back from speaking. While this limited verbal expression may initially reflect an adaptive response to threat rather than a lack of ability [65], over time, reduced opportunities for expression may limit practice and feedback, further slowing expressive language development and may reduce confidence in communicating.

These adversities faced by the mother can also have indirect effects through their influence on maternal mental health and the home environment. Increased maternal depressive symptoms associated with violence or other stressors [66, 67], may reduce the quality of cognitive and language stimulation available at home [65]. Children of mothers who have experienced such adversities have been found to show less positive engagement, attention, and verbal interaction, suggesting that it is the accumulation of stressors, rather than one single factor, that affects early development [64, 65, 68]. These findings suggest that language delays observed in children of mothers with PPD may partly arise from the combined effects of multiple adversities including IPV, family maltreatment, financial hardships, and stress, which together can shape children’s early developmental trajectories. Similarly, lower maternal education, though unrelated to PPD in this cohort, was independently associated with increased motor delay risk, regardless of maternal PPD, consistent with previous findings [69]. Additional analyses examining associations between these factors and child development are provided in Supplementary File 8.

Contrary to earlier south Asian studies [12, 70], we found no gender differences in the PPD–ECD association. While some evidence suggests boys may be more vulnerable [70, 71], including long-term delays in low-SES contexts [12, 16], our findings align with studies reporting comparable risk across genders [72]. These mixed patterns highlight the need for continued investigation.

### Limitations

Our findings should be considered alongside several important limitations. First, the longitudinal nature of the analysis means influence of key unmeasured confounders, such as change in household circumstances, income, or social support over time, cannot be ruled out. However, embedding within a cluster-randomized controlled trial provided methodological strengths, standardized data collection and adjustment for trial arm allocation and clustering, which enhance internal validity. Moreover, since this analysis focused on risk factors and associations, interpretations are independent of the intervention. Second, the analytic sample for examining PPD–ECD associations comprised 1250 mother–child dyads, smaller than the overall PPD sample due to predefined trial timelines based on child birth dates. As inclusion was independent of maternal or child outcomes, the risk of systematic bias was minimised, and statistical power remained adequate. Moreover, PPD risk factor models re-estimated within this subsample, yielded results consistent with the full maternal cohort, suggesting that smaller analytic sample did not substantially bias the observed associations. Use of a whole-population sample within trial clusters further strengthens representativeness and reduces selection bias, enhancing internal validity in routine rural settings. Third, while PPD and child development were measured at two separate time points (12 and 18 months, respectively), each was assessed only once. This limited our ability to examine symptom trajectories or distinguish between persistent and newly emergent depressive episodes. Fourth, the absence of variables including antenatal depression, prior history of depression, co-occurring anxiety, quality of home environment, constrains a more nuanced understanding of PPD risks and potential PPD-ECD pathways. Finally, while findings from this rural Indian setting may not reflect urban populations, they remain directly applicable to similar low-resource rural settings and contribute much-needed evidence from an underrepresented region in global maternal mental health research.

## Conclusion

To our knowledge, this is the first study from rural India to examine how PPD at 12 months, an overlooked period in maternal mental health, relates to child development at 18 months, with language delays showing particular vulnerability. Maternal adversity and socioeconomic disadvantage emerged as key contributors to PPD. While associations between PPD and language delays attenuated after adjustment, this multi-level analysis provides deeper insight into how underlying adversities contribute to PPD, which may in turn function as a risk factor for ECD. These dynamics are complex with PPD’s relationship with child development being unlikely to be uniform. Our findings therefore suggest that PPD may not be a standalone condition but part of a broader pattern of compromised maternal wellbeing within the wider ecosystem of maternal and early child development. These insights underscore the need to monitor maternal mental health beyond the early postpartum phase, particularly during the 12–18-month window, when unaddressed maternal vulnerabilities may have lasting developmental impacts, but remain largely neglected in India’s health system. These findings carry important implications for both research and practice, as discussed below.

### Implications for Indian and similar resource-limited rural health systems

India has no dedicated national policy on maternal mental health, despite the high burden of perinatal mental disorders. While some states have promising initiatives, most women lack systematic support. This policy gap highlights the need for integrated solutions within the rural health system. First, the findings of the study point to several opportunities for strengthening support for PPD within India’s rural health system and similar low-resource settings. Existing frontline workers, Accredited Social Health Activists (ASHAs) and Anganwadi Workers (AWWs), operating under the National Rural Health Mission [73] and the Integrated Child Development Scheme [74], offer a strong foundation for household-level engagement. Based on the key risk factors identified in this study, there is scope to develop a simple PPD risk profile grounded in commonly observed indicators of maternal adversity and disadvantage. Such a profile could support targeted screening and early intervention within existing health systems.

In parallel, coordination with the National and District Mental Health Programs (NMHP and DMHP) [75] could enable more integrated care. ASHAs and AWWs, embedded in communities through routine contact, are well placed to identify early psychosocial risks. DMHP-trained community health workers (CHWs), who already conduct home assessments for severe mental illness, could expand their role to maternal mental health. This programmatic coordination could enhance PPD detection, enable timely responses, and improve referral pathways. These insights also have implications for strengthening support within frontline services. Training ASHAs, AWWs and CHWs to recognise maternal distress and offer low-intensity emotional and practical support within their existing roles could provide a scalable approach. This could further be effective when complemented by non-governmental and community-based initiatives that integrate maternal mental health into routine care. The domain-specific association between PPD and language delays also highlights the value of incorporating brief, validated language screening tools into existing growth monitoring protocols.

Future implementation research should explore how culturally embedded expressions of PPD, caregiving practices, and shifting gender norms shape maternal mental health. Integrating an understanding of these factors alongside the distinct PPD risk profiles, will be key to designing feasible, contextually grounded interventions in rural LMIC settings.

## Supplementary Information

Supplementary Material 1: Supplementary File 1_SPRING Intervention_Brief Description_Original Research_BMC Psychology_Kumar D.docx.

Supplementary Material 2: Supplementary File 2_Analytical Framework_Original Research_BMC Psychology_Kumar D.docx.

Supplementary Material 3: Supplementary File 3_Cronbach's Alpha Original Research_BMC Psychology_Kumar D.docx.

Supplementary Material 4: Supplementary File 4_Detailed Flowchart_Original Research_BMC Psychology_Kumar D.docx.

Supplementary Material 5: Supplementary File 5_Descriptive characteristics of mothers_Original Research_BMC Psychology_Kumar D.docx.

Supplementary Material 6: Supplementary File 6_Individual maternal adverse events_Original Research_BMC Psychology_Kumar D.docx.

Supplementary Material 7: Supplementary File 7_Graphs_Original Research_BMC Psychology_Kumar D.docx.

Supplementary Material 8: Supplementary File 8_Additional analysis_Association Risk Factors and Child Development_Original Research_BMC Psychology_Kumar D.docx.

## Data Availability

The study datasets supporting the conclusions of this article are available in these online repositories. Their names and accession number(s) can be found at: World Bank International Household Survey Network (IHSN) microdata catalogue (https://catalog.ihsn.org/catalog/7952/study-description) [76], LSHTM Data Compass data repository (https://doi.org/10.17037/DATA.00003124 and https://doi.org/10.17037/DATA.00000947) [77, 78]. The Indian dataset includes outcomes for 12,467 mothers at baseline and endline evaluations. Additionally, trial data is available through the World Bank Microdata Library [https://doi.org/10.48529/4h78-tf43] [79] and https://microdata.worldbank.org/index.php/catalog/5684.

## Abbreviations

ASHA: Accredited Social Health Activist
ANM: Auxiliary Nurse Midwife
AWW: Anganwadi Worker
BSID-III: Bayley Scales of Infant and Toddler Development – Version III
CHW: Community Health Workers
cRCT: Cluster randomised controlled trial
DMHP: District Mental Health Program
DSM-IV: Diagnostic and Statistical Manual -Version IV
ECD: Early child development
IPV: Intimate partner violence
LMIC: Low- and middle- income countries
MAE: Maternal adverse events
NFHS: National Family Health Survey
NMHP: National Mental Health Program
OR: Odds ratio
PHQ-9: Patient Health Questionnaire-9
PPD: Postpartum depression
RRR: Relative risk ratio
SES: Socio-economic status
SPRING: Sustainable PRogramme Incorporating Nutrition and Games
UK: United Kingdom
