# Supplementary material for: Unpacking postpartum depression in rural India: an integrated analysis of risk factors at 12 months and child development outcomes at 18 months of age – findings from the SPRING study
Source: BMC Psychol. 2026 Jan 19;14:79. doi: 10.1186/s40359-025-03746-1 (PMC12817435; doi:10.1186/s40359-025-03746-1)
Supplement: Supplementary file 1 — Supplementary Material 1: Supplementary File 1_SPRING Intervention_Brief Description_Original Research_BMC Psychology_Kumar D.docx. [file 40359_2025_3746_MOESM1_ESM.docx]

**Supplementary File 1: Brief Description of the SPRING Intervention**

**Sustainable PRogramme Incorporating Nutrition and Games (SPRING) Intervention**

SPRING was designed as an innovative, scalable home-visiting programme delivered by a newly developed cadre of Community-Based Agents (CBAs) from pregnancy through the first two years of life, aiming to maximise child development, growth and survival [1,2]. It was adapted from the WHO/UNICEF Care for Child Development package [3] . The intervention’s change pathway hypothesised that child outcomes would be directly improved through enhanced feeding, interaction and play behaviours, and indirectly through improved maternal caregiving, mental health, well-being and efficacy [3–5].

SPRING employed a supportive counselling approach adapted from cognitive behavioural therapy (CBT), previously shown effective in Pakistan [6], and built on the five pillars of maternal psychosocial wellbeing [7]. Structured strategies based on CBT principles were adapted for delivery by non-specialist CBAs. While not targeting depressive symptoms directly, the intervention aimed to strengthen maternal well-being by enhancing caregiving practices and family support. Through behavioural activation, CBAs helped mothers break down daily caregiving tasks—feeding, play, and stimulation—into achievable steps using available resources, thereby building mastery and improving mood. Problem-solving techniques supported mothers in identifying barriers, exploring solutions, and involving family members.

The CBA–mother relationship, often perceived as peer-like, was another source of support, aimed at reducing isolation and fostering trust. Regular home visits with empathic listening created safe spaces for mothers to talk, reflect and attempt small behavioural changes. Other elements included guided discovery with counselling cards, modelling through coaching and demonstration, and praise to reinforce maternal effort [1]. Collectively, these strategies aimed to build confidence and coping, addressing psychosocial stressors that may contribute to low mood.

The intervention was delivered to all 990 mother in the intervention arm (mothers in control arms = 1017), regardless of their PPD status using a universal prevention approach. It was not targeted specifically at women identified as high risk or already experiencing elevated depressive symptoms.

**References:**

1. Kirkwood BR, Sikander S, Roy R, Soremekun S, Bhopal SS, Avan B, et al. Effect of the SPRING home visits intervention on early child development and growth in rural India and Pakistan: parallel cluster randomised controlled trials. Front Nutr. Frontiers Media SA; 2023;10. https://doi.org/10.3389/fnut.2023.1155763
2. Hill Z, Zafar S, Soremekun S, Sikander S, Avan BI, Roy R, et al. Can home visits for early child development be implemented with sufficient coverage and quality at scale? Evidence from the SPRING program in India and Pakistan. Front Nutr. Frontiers Media SA; 2023;10. https://doi.org/10.3389/fnut.2023.1152548
3. UNICEF, WHO. Care for Child Development Package [Internet]. 2015. https://www.unicef.org/documents/care-child-development
4. Yousafzai AK, Rasheed MA, Rizvi A, Armstrong R, Bhutta ZA. Effect of integrated responsive stimulation and nutrition interventions in the Lady Health Worker programme in Pakistan on child development, growth, and health outcomes: a cluster-randomised factorial effectiveness trial. The Lancet. 2014;384:1282–93. https://doi.org/10.1016/S0140-6736(14)60455-4
5. UNICEF. Promoting Care for Child Development in Community Health Services: A Summary of the Pakistan Early Child Development Scale-up (PEDS) Trial. 2013 Sep.
6. Rahman A, Malik A, Sikander S, Roberts C, Creed F. Cognitive behaviour therapy-based intervention by community health workers for mothers with depression and their infants in rural Pakistan: a cluster-randomised controlled trial. The Lancet. 2008;372:902–9. https://doi.org/10.1016/S0140-6736(08)61400-2
7. Zafar S, Sikander S, Haq Z, Hill Z, Lingam R, Skordis-Worrall J, et al. Integrating maternal psychosocial well-being into a child-development intervention: The five-pillars approach. Ann N Y Acad Sci. 2014;1308:107–17. https://doi.org/10.1111/nyas.12339
