# Supplementary material for: Unpacking postpartum depression in rural India: an integrated analysis of risk factors at 12 months and child development outcomes at 18 months of age – findings from the SPRING study
Source: BMC Psychol. 2026 Jan 19;14:79. doi: 10.1186/s40359-025-03746-1 (PMC12817435; doi:10.1186/s40359-025-03746-1)
Supplement: Supplementary file 2 — Supplementary Material 2: Supplementary File 2_Analytical Framework_Original Research_BMC Psychology_Kumar D.docx. [file 40359_2025_3746_MOESM2_ESM.docx]

**Supplementary File 2**

**Analytical framework: Risk factors for postpartum depression (PPD) and association between PPD and child development outcomes**

**Demographic-economic**

**Lack of social support; high stress**

**Maternal adverse life events**

**RISK FACTORS**

**PPD at 12 months postpartum**

**CHILD DEVELOPMENT**

**At 18 Months of age**

**PPD: postpartum depression*

*The model depicts three key contextual risk factors – demographic-economic disadvantage, limited social support with high stress, and maternal adverse life events – that are proposed to contribute to PPD at 12 months postpartum in rural India. It further illustrates how maternal PPD is associated with language, motor, and cognitive development in children at 18 months of age, and how the identified risk factors influence these associations. Solid arrows represent direct associations between exposures and outcomes, while dashed arrows indicate the influence of other variables.*
