# Supplementary material for: Unpacking postpartum depression in rural India: an integrated analysis of risk factors at 12 months and child development outcomes at 18 months of age – findings from the SPRING study
Source: BMC Psychol. 2026 Jan 19;14:79. doi: 10.1186/s40359-025-03746-1 (PMC12817435; doi:10.1186/s40359-025-03746-1)
Supplement: Supplementary file 3 — Supplementary Material 3: Supplementary File 3_Cronbach's Alpha Original Research_BMC Psychology_Kumar D.docx. [file 40359_2025_3746_MOESM3_ESM.docx]

# Supplementary File 3:

**Table 5: Cronbach’s Alpha for maternal and child development measures in this paper’s analyses**

| Measure | Subscale | Cronbach’s α |
| --- | --- | --- |
| Patient Health Questionnaire-9 (PHQ-9) | Total | 0.74 |
| Bayley Scales of Infant and Toddler Development, Third Edition (BSID-III) | Expressive language | 0.79 |
|  | Receptive language | 0.79 |
|  | Cognitive | 0.67 |
|  | Gross motor | 0.92 |
|  | Fine motor | 0.68 |
| DUKE’s Social Support and Stress Scale | Social support | 0.65 |
|  | Social stress | 0.77 |
| Maternal adversity index | Total | 0.78 |
