# Supplementary material for: Unpacking postpartum depression in rural India: an integrated analysis of risk factors at 12 months and child development outcomes at 18 months of age – findings from the SPRING study
Source: BMC Psychol. 2026 Jan 19;14:79. doi: 10.1186/s40359-025-03746-1 (PMC12817435; doi:10.1186/s40359-025-03746-1)
Supplement: Supplementary file 4 — Supplementary Material 4: Supplementary File 4_Detailed Flowchart_Original Research_BMC Psychology_Kumar D.docx. [file 40359_2025_3746_MOESM4_ESM.docx]

**Supplemental File 4**

**Figure 2. Participant flow for maternal (12 months) plus child development assessments (18 months) within the SPRING trial**

24 clusters randomised

| Intervention |
| --- |
| 12 clusters |

| Control |
| --- |
| 12 clusters |

| Mothers | **2535** |
| --- | --- |
| Children | **2554** |

| Mothers | **2543** |
| --- | --- |
| Children | **2563** |

**Livebirths from 18 June 2015 identified by surveillance system**

| **0.7%** | **Excluded** |
| --- | --- |
| Child not living with mother | |
| Mother – incapable of assessment | |
| Child – major congenital defect | |

| **Excluded** | **0.7%** |
| --- | --- |
| Child not living with mother | |
| Mother – incapable of assessment | |
| Child – major congenital defect | |

**Eligible maternal + child development sample**

| Mothers **^b^** | **1300** |
| --- | --- |
| Children | **864** |

| Mothers **^a^** | **1309** |
| --- | --- |
| Children | **862** |

| **11.7%** | **Lost to follow-up (12 months)** |
| --- | --- |
| Mother death | |
| Child death | |
| Moved away | |
| Consent refused | |

| **Lost to follow up (12 months)** | **8.8%** |
| --- | --- |
| Mother death | |
| Child death | |
| Moved away | |
| Consent refused | |

| **Mothers available for 12 months assessment** | **1187** | **90.7%** |
| --- | --- | --- |
| **Assessments Done** | **1017** | **85.7%** |
| No appointment possible | | |
| Mother/child ill on appointment day | | |
| Appointment taken but not done on the day | | |

| **87.5%** | **1138** | **Mothers available for 12 months assessment** |
| --- | --- | --- |
| **87.1%** | **991^c^** | **Assessments Done** |
| No appointment possible | | |
| Mother/child ill on appointment day | | |
| Appointment taken but not done on the day | | |

| **BSID-III not done** | **387^d^** | **38.1%** |
| --- | --- | --- |
| ***Reason:*** *Trial’s pre-specified PPD sample size > ECD sample size* | | |

| **38.4%** | **381^d^** | **BSID-III not done** |
| --- | --- | --- |
| ***Reason:*** *Trial’s pre-specified PPD sample size > ECD sample size* | | |

| **Mother-baby dyads:** 12 months PHQ-9 and 18 months BSID-III assessments done | **635 ^d^** | **62.4%** |
| --- | --- | --- |

| **62.1%** | **615 ^d^** | **Mother-baby dyads:** 12 months PHQ-9 and 18 months BSID-III assessments done |
| --- | --- | --- |

^a, b^ Eligible mothers for 12M assessments once sample size requirement was met. **^c^** PHQ-9 not done for one mother. ^d^ These numbers include twins as BSID-III was administered with both the twins

Source:

1. Kirkwood BR, Sikander S, Roy R, Soremekun S, Bhopal SS, Avan B, et al. Effect of the SPRING home visits intervention on early child development and growth in rural India and Pakistan: parallel cluster randomised controlled trials. Front Nutr. 2023;10. <https://doi.org/10.3389/fnut.2023.1155763>
2. Kumar D, Soremekun S, Roy R, Verma D, Bhopal S, Sharma KK, Divan G, Kirkwood BR and Avan BI. Impact of SPRING, an Integrated Mother–Child Focused Psychosocial Home-visiting Intervention, on Postpartum Depression in Rural India: A Cluster Randomized Controlled Trial. Indian J Psychol Med. 2025;1–10.
