# Supplementary material for: Unpacking postpartum depression in rural India: an integrated analysis of risk factors at 12 months and child development outcomes at 18 months of age – findings from the SPRING study
Source: BMC Psychol. 2026 Jan 19;14:79. doi: 10.1186/s40359-025-03746-1 (PMC12817435; doi:10.1186/s40359-025-03746-1)
Supplement: Supplementary file 5 — Supplementary Material 5: Supplementary File 5_Descriptive characteristics of mothers_Original Research_BMC Psychology_Kumar D.docx. [file 40359_2025_3746_MOESM5_ESM.docx]

**Supplementary File 5**

**Table 6: Descriptive Characteristics of Mothers with PHQ-9 Data: Included versus Excluded from PPD-ECD Association Analysis**

| Indicators | With PHQ-9 data, included vs excluded from analysis | | |
| --- | --- | --- | --- |
|  | **Total mothers** | **Included (I)** | **Excluded (E)** |
|  | **2018^d^** | **1250** | **768^d^** |
| *n* Mothers with ≤5 years of education ^a^ (%) | 1795  (88.99%) | 1101  (88.08%) | 694^e^  (90.48%) |
| *n* Mothers belonging to lowest quintile ^b^ (%) | 412  (20.42%) | 268  (21.44%) | 144  (18.75%) |
| *n* Mothers belonging to less privileged caste ^c^ (%) | 1236  (61.25%) | 751  (60.08%) | 485  (63.15%) |
| *n* Mothers delivering a female child (%) | 1115  (55.25%) | 666  (53.28%) | 449  (58.46%) |
| *n* Twins/Triplets (%) | 28  (1.39%) | 20  (1.60%) | 8  (1.04%) |
| *n* Delivery in non-facility (%) | 38  (1.88%) | 22  (1.76%) | 16  (2.08%) |
| *Mean* maternal age at birth of the index child (sd) | 22.33  (3.83) | 22.34  (3.77) | 22.31  (3.94) |
| ^a^ Maternal education: 0 = <5 years of education, 1 = ≥5 years of education completed.  ^b^ Mother’s socio-economic status (SES): In quintiles (1–5); 1 = poorest (bottom 20%), 5 = least poor (top 20%)  ^c^ 0 = general category; 1 = scheduled caste, scheduled tribe or other backward class (SC/ST/OBC)  ^d^ These numbers include twins as BSID-III was administered with both the twins.  ^e^ Data unavailable for one mother on education.  PPD: Postpartum depression; ECD: Early child development | | | |
