# Supplementary material for: Unpacking postpartum depression in rural India: an integrated analysis of risk factors at 12 months and child development outcomes at 18 months of age – findings from the SPRING study
Source: BMC Psychol. 2026 Jan 19;14:79. doi: 10.1186/s40359-025-03746-1 (PMC12817435; doi:10.1186/s40359-025-03746-1)
Supplement: Supplementary file 6 — Supplementary Material 6: Supplementary File 6_Individual maternal adverse events_Original Research_BMC Psychology_Kumar D.docx. [file 40359_2025_3746_MOESM6_ESM.docx]

**Supplementary File 6**

**Table 7: Individual Maternal Adverse Events Associated with PPD at 12 Months Postpartum**

| Risk Factors |  | Total  n  (%) | With PPD  n  (%) | Without PPD  n  (%) | OR  (95% CI) | p-value |
| --- | --- | --- | --- | --- | --- | --- |
|  |  | 1250* | 230 | 1020 |  |  |
| Mother ill during pregnancy and postpartum | **Yes** | 108  (8.64%) | 40  (17.39%) | 68  (6.67%) | **2.89**  (1.87, 4.47) | **<0.001** |
|  | **No** | 1142  (91.36%) | 190  (82.61%) | 953  (93.33%) |  |  |
| Any close family member ill/injured postpartum | **Yes** | 479  (38.32%) | 102  (44.35%) | 377  (36.96%) | 1.30  (0.96, 1.75) | 0.084 |
|  | **No** | 771  (61.68%) | 128  (55.65%) | 643  (63.04%) |  |  |
| Problems caused by husband’s alcohol? | **Yes** | 105  (8.40%) | 25  (10.87%) | 80  (7.84%) | 1.41  (0.86, 2.29) | 0.163 |
|  | **No** | 1145  (91.60%) | 205  (89.13%) | 940  (92.16%) |  |  |
| Physical abuse /violence by husband | **Yes** | 89  (7.12%) | 36  (15.65%) | 53  (5.20%) | **3.43**  (2.14, 5.45) | **<0.001** |
|  | **No** | 1161  (92.88%) | 194  (84.35%) | 967  (94.80%) |  |  |
| Verbal abuse by husband | **Yes** | 82  (6.56%) | 32  (13.91%) | 50  (4.90%) | **3.05**  (1.88, 4.95) | **<0.001** |
|  | **No** | 1168  (93.44%) | 198  (86.09%) | 970  (95.10%) |  |  |
| Mistreatment of mother by family and others | **Yes** | 73  (5.84%) | 31  (13.48%) | 42  (4.12%) | **3.46**  (2.09, 5.73) | **<0.001** |
|  | **No** | 1177  (94.16%) | 199  (86.52%) | 978  (95.88%) |  |  |
| Family in debt since pregnancy | **Yes** | 214  (17.12%) | 62  (26.96%) | 152  (14.90%) | **2.03**  (1.43, 2.87) | **<0.001** |
|  | **No** | 1036  (82.88%) | 168  (73.04%) | 868  (85.10%) |  |  |
| Maternal adverse events: A cumulative index of adverse life events experienced by mothers was constructed by summing seven binary indicators (listed above), each capturing a distinct domain of maternal adversity. These were assessed using items from the Early Life Stress Questionnaire^33^ administered at 12 months postpartum. Each of the seven adverse events was constructed as a sub-score by summing related items, as described below, and then included as a separate component in the final index.  All adverse events were reported as occurring during pregnancy or the postpartum period, and grouped into the following seven components:   1. Serious maternal illness, based on reports of illness during pregnancy and after childbirth; 2. Illness or death of a close family member, created by summing reports across multiple individuals, including parent, sibling, child, or other relatives; 3. Husband’s alcohol-related problems, as reported by the mother (single item); 4. Physical abuse or violence by the husband, created by summing nine items including slapping, pushing, hitting with a fist, kicking, choking, assault with a weapon, and three items on forced sexual activity; 5. Verbal abuse by the husband, created by summing four behaviours: insulting, belittling, intimidating, and threatening; 6. Mistreatment by other family members, created by summing items capturing physical, sexual, or other forms of abuse or mistreatment by specified individuals; 7. Family indebtedness, reported as having occurred since the pregnancy (single item).   Each component was coded as 0 (absent) or 1 (present), yielding a total index score ranging from 0 to 7. This cumulative score was treated as a continuous predictor in the regression models to examine risk factors for PPD at 12 months and to account for potential confounding effects in models investigating PPD and early child development outcomes.  * Stressful life events were assessed in a subsample of mothers (fewer than those with PHQ-9 data); all 1,250 had PHQ-9 assessments at 12 months and BSID-III at 18 months.  PPD: Postpartum depression | | | | | | |
