# Supplementary material for: Unpacking postpartum depression in rural India: an integrated analysis of risk factors at 12 months and child development outcomes at 18 months of age – findings from the SPRING study
Source: BMC Psychol. 2026 Jan 19;14:79. doi: 10.1186/s40359-025-03746-1 (PMC12817435; doi:10.1186/s40359-025-03746-1)
Supplement: Supplementary file 7 — Supplementary Material 7: Supplementary File 7_Graphs_Original Research_BMC Psychology_Kumar D.docx. [file 40359_2025_3746_MOESM7_ESM.docx]

**Supplementary File 7: Graphs**

**Graph 1. Relationship between overall PHQ-9 scores and maternal adverse events index**

0 1 2 3 4 5 6

**Graph 2. Relationship between PHQ9 scores and SES Quintiles**
