# Supplementary material for: Unpacking postpartum depression in rural India: an integrated analysis of risk factors at 12 months and child development outcomes at 18 months of age – findings from the SPRING study
Source: BMC Psychol. 2026 Jan 19;14:79. doi: 10.1186/s40359-025-03746-1 (PMC12817435; doi:10.1186/s40359-025-03746-1)
Supplement: Supplementary file 8 — Supplementary Material 8: Supplementary File 8_Additional analysis_Association Risk Factors and Child Development_Original Research_BMC Psychology_Kumar D.docx. [file 40359_2025_3746_MOESM8_ESM.docx]

**Supplementary File 8**

**Table 8: Additional analyses examining associations between risk factors and child development**

| Language delays at 18 months of child’s age | | | | |
| --- | --- | --- | --- | --- |
| Mild to moderate delay (-1SD) | **RRR** | **P-value** | **[95% conf. interval]** | |
| Mother’s socio-economic status (SES) | 0.84 | 0.008 | 0.74 | 0.95 |
| Maternal education | 0.74 | 0.223 | 0.46 | 1.19 |
| Gender of the child | 0.87 | 0.402 | 0.64 | 1.19 |
| Maternal adverse events (MAE) | 1.21 | 0.005 | 1.06 | 1.38 |
| Severe delay (-2SD) | **RRR** | **P-value** | **[95% conf. interval]** | |
| Mother’s socio-economic status (SES) | 0.73 | 0.000 | 0.62 | 0.87 |
| Maternal education | 0.52 | 0.018 | 0.30 | 0.89 |
| Gender of the child | 0.80 | 0.281 | 0.54 | 1.19 |
| Maternal adverse events (MAE) | 1.03 | 0.711 | 0.87 | 1.23 |
| Motor delays at 18 months of child’s age | | | | |
| Mild to moderate delay (-1SD) | **RRR** | **P-value** | **[95% conf. interval]** | |
| Mother’s socio-economic status (SES) | 0.87 | 0.117 | 0.73 | 1.04 |
| Maternal education | 0.47 | 0.007 | 0.27 | 0.81 |
| Gender of the child | 0.89 | 0.617 | 0.59 | 1.37 |
| Maternal adverse events (MAE) | 1.13 | 0.167 | 0.95 | 1.35 |
| Severe delay (-2SD) | **RRR** | **P-value** | **[95% conf. interval]** | |
| Mother’s socio-economic status (SES) | 0.82 | 0.335 | 0.55 | 1.22 |
| Maternal education | 0.21 | 0.001 | 0.08 | 0.55 |
| Gender of the child | 0.72 | 0.453 | 0.29 | 1.71 |
| Maternal adverse events (MAE) | 1.37 | 0.045 | 1.006 | 1.85 |
| Cognitive delays at 18 months of child’s age | | | | |
| Mild to moderate delay (-1SD) | **RRR** | **P-value** | **[95% conf. interval]** | |
| Mother’s socio-economic status (SES) | 0.81 | 0.003 | 0.69 | 0.93 |
| Maternal education | 0.89 | 0.662 | 0.53 | 1.48 |
| Gender of the child | 1.16 | 0.378 | 0.83 | 1.63 |
| Maternal adverse events (MAE) | 0.97 | 0.662 | 0.82 | 1.13 |
| Severe delay (-2SD) | **RRR** | **P-value** | **[95% conf. interval]** | |
| Mother’s socio-economic status (SES) | 0.88 | 0.400 | 0.65 | 1.18 |
| Maternal education | 0.45 | 0.090 | 0.18 | 1.13 |
| Gender of the child | 0.82 | 0.588 | 0.39 | 1.69 |
| Maternal adverse events (MAE) | 1.23 | 0.136 | 0.93 | 1.63 |
| RRR: Relative Risk Ratio  Developmental Delay: -1SD of global average (composite score ≥70 and <85 on BSID-III); -2SD of global average (composite score<70 on BSID-III). | | | | |
